# Ethambutol induces impaired autophagic flux and apoptosis in the rat retina

**DOI:** 10.1242/dmm.019737

**Published:** 2015-08-01

**Authors:** Shun-Ping Huang, Jia-Ying Chien, Rong-Kung Tsai

**Affiliations:** 1 Department of Molecular Biology and Human Genetics, Tzu Chi University, Hualien 97002, Taiwan; 2 Institute of Eye Research, Buddhist Tzu Chi General Hospital, Hualien 97002, Taiwan; 3 Institute of Medical Sciences, Tzu Chi University, Hualien 97002, Taiwan

**Keywords:** Ethambutol, Optic neuroretinopathy, Autophagy, Rottlerin, Apoptosis

## Abstract

Ethambutol (EMB), an effective first-line antituberculosis agent, can cause serious visual impairment or irreversible vision loss in a significant number of patients. However, the mechanism underlying this ocular cytotoxicity remains to be elucidated. In this study, we found that there were statistically significant dose- and time-dependent increases in the number of cytoplasmic vacuoles and the level of cell death in EMB-treated RGC-5 cells (retinal ganglion cells). The protein kinase C (PKC)δ inhibitor rottlerin markedly reduced the EMB-induced activation of caspase-3 and the subsequent apoptosis of RGC-5 cells. Western blot analysis revealed that the expression levels of class III PI3K, Beclin-1, p62 and LC3-II were upregulated, and LC3 immunostaining results showed activation of the early phase and inhibition of the late stage of autophagy in retinas of the EMB-intraperitoneal (IP)-injected rat model. We further demonstrated that exposure to EMB induces autophagosome accumulation, which results from the impaired autophagic flux that is mediated by a PKCδ-dependent pathway, inhibits the PI3K/Akt/mTOR signaling pathway and leads to apoptotic death in retina neuronal cells. These results indicate that autophagy dysregulation in retinal neuronal cells might play a substantial role in EMB-induced optic neuroretinopathy.

## Abstract (precis)

**Summary:** This study provides the first evidence that EMB induces autophagosome accumulation, which results from the impaired autophagic flux that is mediated by a PKCδ-dependent pathway, and leads to apoptotic death in retina neuronal cells.

## INTRODUCTION

Ethambutol (EMB) is routinely used as an anti-mycobacterial agent, especially in the treatment of tuberculosis. EMB can cause an irreversible vision loss, known as EMB-induced optic neuropathy (EON), in a small but significant fraction of patients. Depending on the dosage of EMB, the incidence of EON has been reported to be in the range of 1-5% (Sivakumaran et al., 1998). It has been suggested that the cause of EON might be linked to a disturbance in the optic nerve that is induced by EMB through an excitotoxicity pathway (Campbell and Elmes, 1975; Chai and Foroozan, 2007; Figueroa et al., 1971; Heng et al., 1999; Yoon et al., 2000; Zoumalan et al., 2005). The toxic effects of EMB in retinal cells have also been studied in recent works (Chung et al., 1989; Liu et al., 2008; Tsai et al., 2008a; Vistamehr et al., 2007). The clinical features of EMB-induced neuroretinopathy, including macula edema, retinal hemorrhage, retinal pigment epithelial (RPE) dysfunction and electrophysiological abnormalities, have also been reported (Liu et al., 2008; Vistamehr et al., 2007; Yen et al., 2000) but at lower frequencies than EON. Although the visual impairment is generally reversible after discontinuation of EMB, some patients still suffer severe or permanent vision loss, even taking the standard doses of EMB (Alvarez and Krop, 1993; DeVita et al., 1987). The pathophysiology of EMB-induced ocular toxicity remains unclear. Our previous studies have demonstrated that EMB induces cytosolic vacuoles formation and reduces the phagocytic activity in human RPE-derived cells, including RPE50 and ARPE19 cells (Tsai et al., 2008a). Furthermore, we also found that EMB-induced cytotoxicity and reduced phagocytosis in RPE cells are mediated via the protein kinase C (PKC)δ signaling pathway (Tsai et al., 2011).

PKC is a family of serine/threonine kinases that are involved in different neuronal development events, such as proliferation, differentiation, survival and apoptosis (Wooten, 1999). The involvement of PKCs in apoptosis has been well established. PKCδ was first identified as a substrate of caspase-3, and the proteolytic activation of PKCδ has been directly linked to apoptosis (Basu et al., 2001; Ghayur et al., 1996). As with apoptosis, PKCδ was the first PKC isozyme shown to play an important role in autophagy (Chen et al., 2009; Ozpolat et al., 2007; Pattingre et al., 2009, 2008). It has been suggested that PKCδ induces autophagy activation to protect cells from cell death in response to acute hypoxic stress, but caspase-3-mediated proteolytic cleavage of PKCδ during chronic stress promotes apoptosis to eliminate irreversibly damaged cells (Chen et al., 2008).

Autophagy is an intracellular self-degradative process for breakdown of the cytoplasmic components, eradicating toxic aggregates and damaged organelles and then recycling the essential biomolecules to maintain cellular metabolism and homeostasis (Yang and Klionsky, 2010). Autophagy represents a cytoprotective response against various types of cellular stress (Boya et al., 2005), and its deregulation has been implicated in the pathogenesis of specific diseases such as immune-mediated diseases, cardiovascular diseases, metabolic diseases, cancer and neurodegeneration (Ravikumar et al., 2010). The physiological roles of autophagy in neural function are far from being completely understood (Nixon, 2006; Wong and Cuervo, 2010). Autophagy activation reduces the accumulation of misfolded and aggregated proteins in several neurodegenerative proteinopathies (Pickford et al., 2008; Renna et al., 2010). Conversely, overactivation of autophagy can trigger neuronal cell death following hypoxic/ischemic injury (Ginet et al., 2009; Koike et al., 2008).

In this study, we found that EMB induces apoptosis in retinal ganglion cells (RGCs) and increases latency in the flash visual evoked potential (FVEP) test in rats. We also provide evidence suggesting that EMB-induced apoptosis involves the activation of caspase-3/7 and a process of decreasing autophagic flux, which results in autophagosome accumulation through PKCδ signaling and the PI3K/Akt/mTOR pathway. Our data show that the PKCδ inhibitor rottlerin significantly reduced EMB-induced phosphorylation of PKCδ, upregulation of autophagic markers, caspase-3/7 activity and apoptotic death in RGC-5 cells. Altogether, these findings demonstrate that the diminished autophagic flux induced by EMB might be one of the factors that leads to cytotoxicity in retinal neuronal cells and contributes to the pathogenesis of EMB-induced optic neuroretinopathy.

**TRANSLATIONAL IMPACT**

**Clinical issue**

Ethambutol (EMB), an efficacious antituberculosis agent, can cause irreversible vision loss owing to EMB-induced optic neuropathy (EON) in a small but significant fraction of patients. Depending on the dosage of EMB, EON incidence has been reported to be in the range of 1-5%. EMB-induced neuroretinopathies, including retinal pigment epithelial changes, macular edema, retinal hemorrhage and abnormal electrophysiological properties, have also been reported but at lower frequencies than EON. It has been suggested that the cause of EON might be linked to a disturbance in the optic nerve that is caused by EMB-induced cytotoxicity. Previous work revealed that the EMB toxicity is mediated by zinc, lysosomal membrane permeabilization and mitochondrial-coupling defects in retinal ganglion cells (RGCs). However, the link between EMB toxicity, RGC death and lysosomal or mitochondrial dysfunction remains to be elucidated. Alterations of autophagy, a physiological process that eliminates dysfunctional cytoplasmic components, could be involved.

****Results****

To investigate the role of autophagy regulation in the pathogenesis of EON, the authors used adult rats treated with 40% over the recommended dose of EMB for 10 consecutive days, which induced ocular side effects. The authors provide evidence that EMB induces apoptosis in RGCs and increases the latency of the flash visual evoked potential (which indicates impaired visual function) in rats. Using the mRFP-GFP tandem fluorescent-tagged LC3 assay (which is able to ‘sense’ the presence of autophagosomes and autolysosomes) to study autophagic flux, the authors demonstrate that EMB-induced apoptosis involves a process of decreasing autophagic flux, which results in autophagosome accumulation via activation of the PKCδ signaling and inhibition of the PI3K/Akt/mTOR pathway. The PKCδ inhibitor rottlerin significantly reduced caspase 3/7 activity and apoptotic death in the RGC-5 cell line.

****Implications and future directions****

This study suggests that EMB treatment might impair the fusion of autophagosomes and lysosomes and thus inhibit autophagic flux. These results are consistent with previous studies suggesting that the site of EMB toxic activity is the lysosome. The current results indicate that dysregulation in the autophagy-dependent protein degradation in the lysosomes might contribute to EON. They also expand our understanding of the mechanism underlying EMB cytotoxic effects in the retina and provide new insights into potential molecular players that might be targeted as a protective strategy against EON.

## RESULTS

### EMB induces apoptosis of RGCs and results in visual dysfunction

**Fig. 1.**
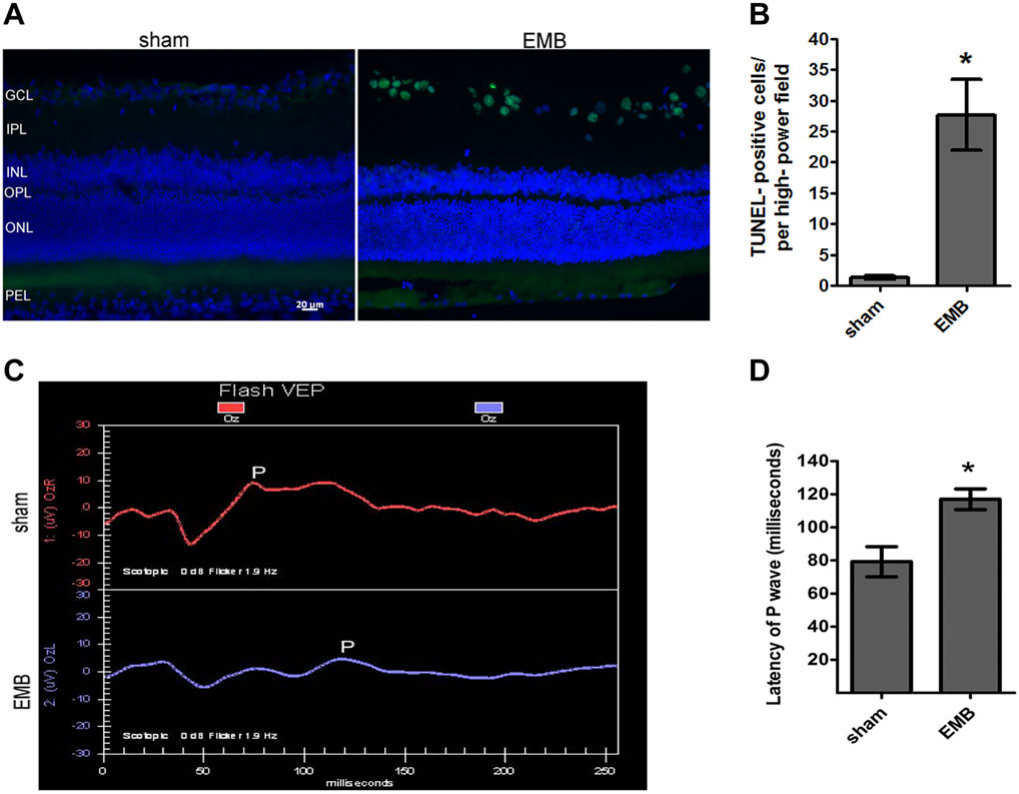
**TUNEL staining of retinas from sham and EMB-treated groups, and FVEP evaluation.** (A) TUNEL-positive apoptotic cells (green) were counted in the GCL under a fluorescence microscope. GCL, ganglion cell layer; IPL, inner plexiform layer; INL, inner nuclear layer; ONL, outer nuclear layer; OPL, outer plexiform layer; PEL, pigmented epithelial layer. Scale bar: 20 µm. (B) The total number of TUNEL-positive cells from six sections from each rat was recorded for the sham and EMB-treated groups, and the average count (mean±s.e.m.) was calculated from three rats from each group. *P<0.05. (C) Representative FVEP tracings after daily EMB IP injections over 10 days. P, P1 wave. (D) Latency of the P1 wave. The latency of P1 in the EMB-treated group (116.9±6.209 ms) was significantly longer compared with the sham group (79.2±9.045 ms). *P<0.05.

The cytotoxicity of EMB in rat retinas was assessed using the terminal deoxynucleotidyl transferase-mediated dUTP nick-end labeling (TUNEL) assay (DeadEnd; Promega, Madison, WI). Compared with the sham group, the number of TUNEL-positive stained cells (Fig. 1A) was significantly higher in the retinal ganglion cell layer (GCL) in the EMB-treated group (*P*<0.05) (Fig. 1B). Furthermore, we evaluated the functional integrity of the visual pathway by assessing the P1 wave latency of the FVEP. In the EMB-treated group, the P1 latency was significantly delayed when compared with the sham group (Fig. 1C,D). The FVEP results demonstrate that the EMB-treated group had significantly impaired visual function compared with the sham group. The data suggest that EMB is toxic to RGCs and leads to impaired electrophysiological functions in the rat retina. These findings are consistent with the reported clinical features of EMB-induced optic neuroretinopathy (Lee et al., 2008; Menon et al., 2009; Woung et al., 1995; Yiannikas et al., 1983).

### Increased levels of autophagic marker and cleaved caspase-3 in EMB-treated retinas

**Fig. 2.**
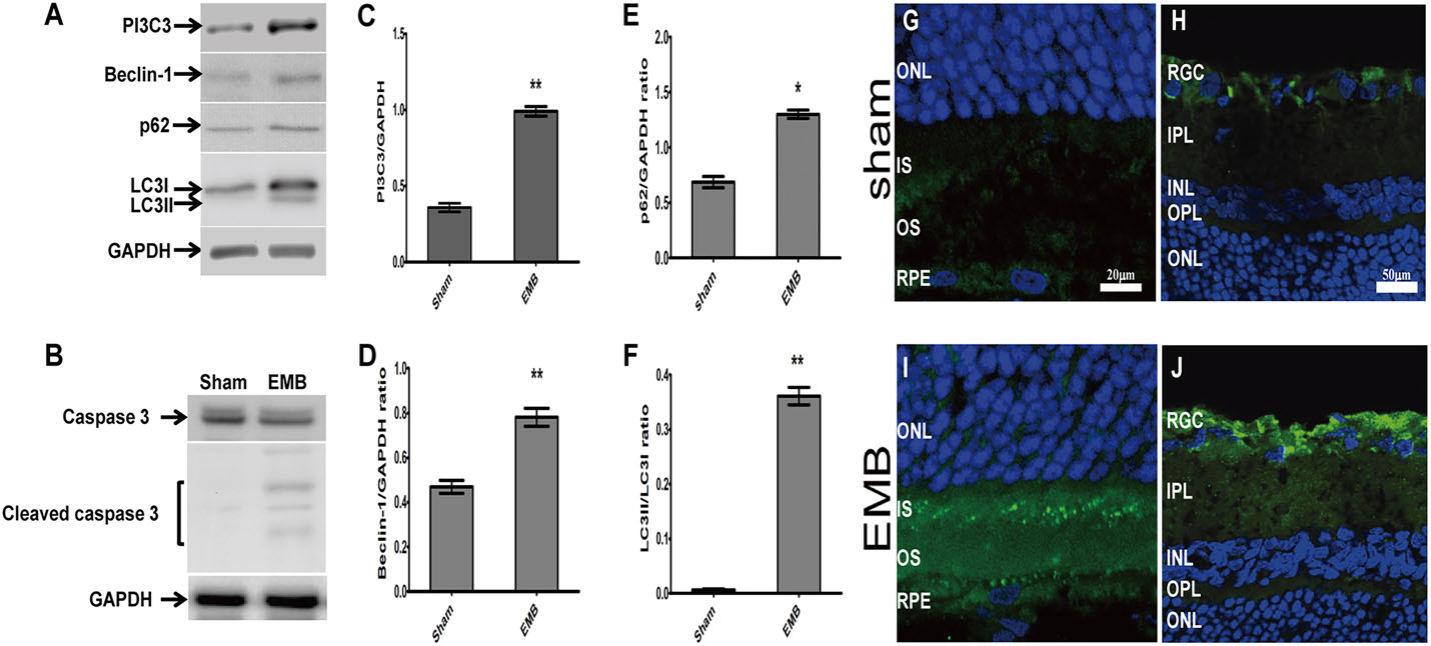
**EMB promotes the expression of cleaved caspase-3 and autophagic markers in the rat retina, and LC3 immunoreactivity is revealed in the rat retina.** (A,B) Immunoblot analysis of the expression levels of cleaved caspase-3 and the autophagic markers PI3C3, Beclin-1, p62 and LC3 in total protein extracts from retinas of the EMB-treated group and the sham group. Representative results from three independent experiments are shown. (C-F) Quantitative analyses of the PI3C3, Beclin-1, p62 and LC3 expression levels normalized to the internal control, GAPDH. The results are expressed as the means±s.e.m. from three independent experiments. *P<0.05, **P<0.01. (G,H) The sham-treated retina showed weak and diffuse staining patterns in the RPE (G) and RGC (H) layers. (I,J) More punctate LC3 fluorescent staining patterns were observed in the IS, RPE (I) and RGC (J) layers of the EMB-treated retina. IS, inner segment; IPL, inner plexiform layer; INL, inner nuclear layer; ONL, outer nuclear layer; OPL, outer plexiform layer; OS, outer segment; RGC, retinal ganglion cell; RPE, retinal pigmented epithelium.

To determine the effect of EMB on autophagy, we investigated the protein levels of class III phosphoinositide 3-kinase (PI3C3; also known as Vps34), beclin-1, p62 and microtubule-associated protein I light chain 3 (LC3) in the retinas of rats treated with EMB or PBS by intraperitoneal (IP) injection. Vps34 and beclin-1 are biochemical markers of autophagy initiation. LC3 is found in two forms in the cell, LC3-I and LC3-II. During autophagosome assembly, LC3 is converted from LC3-I, which is the soluble cytoplasmic form, to LC3-II, which is an LC3-phosphatidylethanolamine conjugate that associates with autophagosomal membranes (Mizushima, 2004). The p62 protein specifically interacts with LC3-II for its degradation by autophagolysosomes, and the cellular level of p62 is negatively correlated with autophagic flux. Western blot analysis revealed that the expression levels of PI3C3, beclin-1 and p62 were upregulated in the EMB-treated group compared with the sham group (Fig. 2A,C-E). An increased LC3-II level was observed in the EMB-treated group but not in the sham group (Fig. 2A,F). These results indicate that EMB promotes the conversion of LC3-I into LC3-II and the accumulation of the proteins PI3C3, beclin-1 and p62. Furthermore, the increase in the p62 protein level together with the increase in the LC3-II level suggests that autophagic flux is impaired in the retinas of the EMB-treated group. To investigate the association between the apoptotic effect of EMB and the autophagy signal, we evaluated the level of cleaved caspase-3, a marker of apoptosis. We found a detectable level of cleaved caspase-3 in the EMB-treated group but no caspase-3 activation in the sham group (Fig. 2B). These findings suggest that the impaired autophagic flux in the EMB-treated group is associated with more autophagic cell death in RGCs.

We further evaluated the expression pattern of LC3 using immunohistochemical staining. In the sham retina, RGCs and RPE cells exhibited a diffuse cytoplasmic LC3 staining pattern (Fig. 2G,H). In the EMB-treated group, the LC3 immunoreactive staining was observed as clusters of intensely stained granules in the RGC and had a punctate appearance in the inner segments of the photoreceptor and RPE cells (Fig. 2I,J). This expression pattern is consistent with the autophagosome formation induced by EMB.

### EMB stimulates PKCδ activation and inhibits the PI3K/Akt/mTOR signaling pathway in the retina

**Fig. 3.**
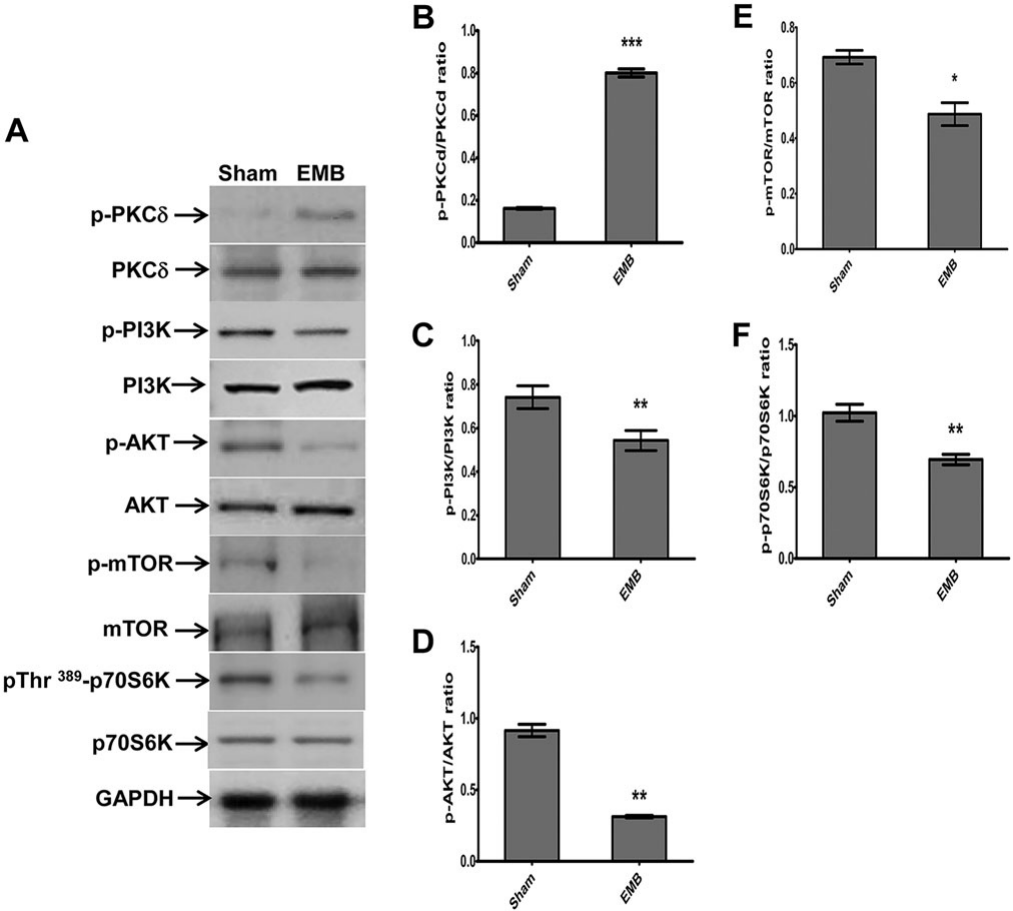
**Effects of EMB on the PKCδ and mTOR-PI3K signaling pathways.** (A) Western blot analysis of the expression levels of PKCδ, phospho-PKCδ, phospho-PI3K, phospho-Akt, phospho-mTOR and phospho-p70S6K in the total protein extracts from retinas of the EMB-treated and sham groups. Representative results from three independent experiments are shown. (B) Quantitative analysis of the PKCδ, PI3K, Akt and mTOR expression levels normalized to the internal control, GAPDH. The results are expressed as the means±s.e.m. from three independent experiments. *P<0.05, **P<0.01, ***P<0.001.

Early activation of PKCδ is involved in hypoxic-stress-induced autophagic responses (Chen et al., 2008). Furthermore, it is well known that the class I phosphatidylinositol 3-phosphate kinase (PI3K)/Akt/mTOR/p70 ribosomal protein S6 kinase (p70S6K) signaling pathway is involved in regulating autophagy. Therefore, we evaluated the effect of EMB on these pathways using western blot analysis. As shown in Fig. 3, there was a significantly higher level of phosphorylated PKCδ (Fig. 3B) in the EMB-treated group compared with the sham group. The levels of phosphorylated mTOR (Fig. 3E) and p70S6K (Fig. 3F) were significantly lower in the EMB-treated group compared with the sham group. To further investigate the upstream inhibition of mTOR by EMB, we investigated the phosphorylation levels of PI3K and Akt. EMB treatment decreased the levels of phosphorylated PI3K (Fig. 3C) and Akt (Fig. 3D) in rat retinas.

### Rottlerin reduces EMB-induced caspase-3 activity and apoptosis in RGC-5 cells

**Fig. 4.**
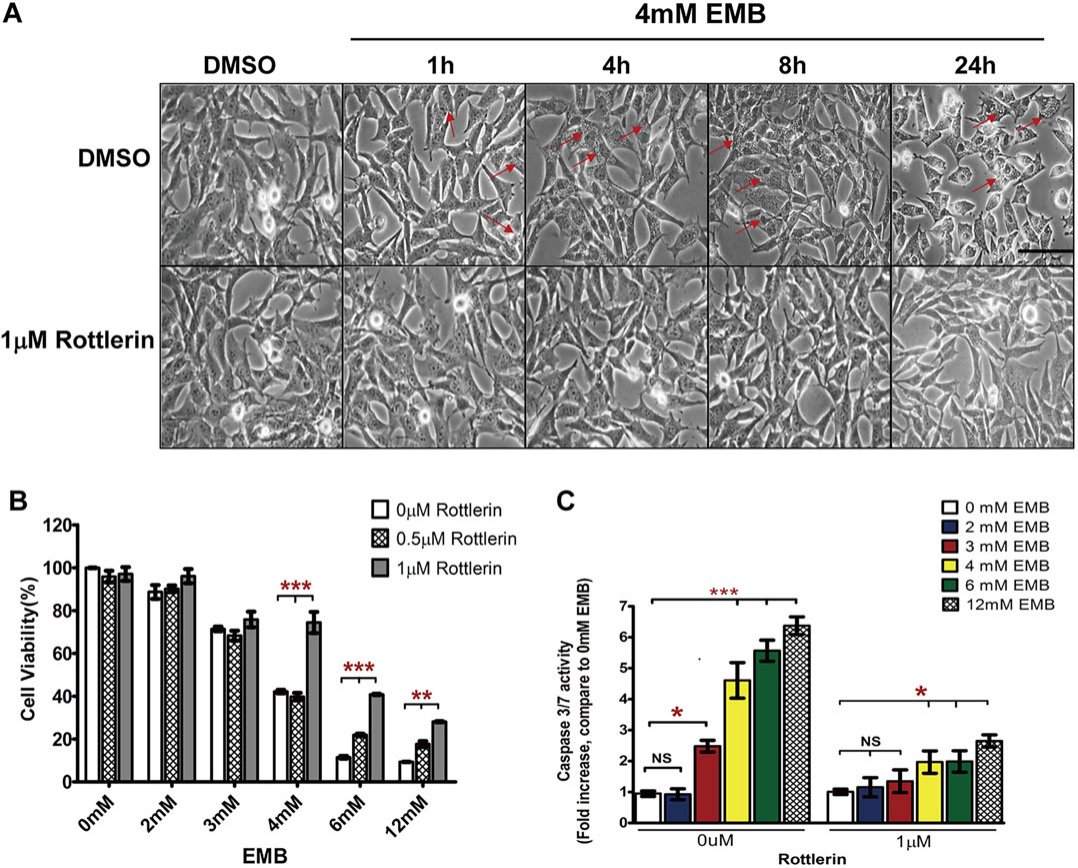
**The PKCδ inhibitor rottlerin attenuates EMB-induced vacuolar formation, caspase-3/7 activation and cell death in RGC-5 cells.** (A,B) EMB-induced cytoplasmic vacuole formation (red arrow) occurs in a time- and dose-dependent manner. EMB treatment (4 mM) resulted in ∼60% RGC-5 cell death, whereas 6 mM and 12 mM EMB treatments were very toxic to the cells, resulting in the death of 80 to 90% cells. The PKCδ inhibitor rottlerin protected the RGC-5 cells from EMB-induced vacuole accumulation and cell death. (C) Caspase-3/7 activity was significantly higher in cultures exposed to 4 to 12 mM EMB for 8 h (*P*<0.001 compared with the DMSO control; *n*=6). No significant increase in caspase-3/7 activity was observed in cultures incubated with 1 µM rottlerin. *P<0.05, **P<0.01, ***P<0.001.

As shown in Fig. 4 and supplementary material Fig. S1, EMB treatment induced cytoplasmic vacuole formation in RGC-5 cells, with the number and diameter of the vacuoles increasing as the time and dosage increased. To evaluate the cell damage caused by EMB, cell viability was determined using the 3-[4,5-dimethylthiazol-2-yl]-2,5 diphenyl tetrazolium bromide (MTT) assay. EMB treatment decreased the RGC-5 viability in a dose-dependent manner (Fig. 4A,B). There was no significant difference in the reduction in viability at the 2 mM concentration (95.3±0.56%) between the EMB-treated cells and the control cells. However, the 3 mM (72.0±0.65%) and 4 mM (42.1±0.8%) EMB concentrations significantly reduced the cell viability of the EMB-treated cells compared with the control cells (*P*<0.001). The 6 mM (11.5±0.62%) and 12 mM (9.3±0.7%) concentrations resulted in reductions in cell viability that were considered beyond the LD_50_. Because the 4 mM EMB treatment resulted in approximately 50 to 60% cell death, this concentration was used in the subsequent experiments. In the presence of EMB, 1 µM rottlerin, a PKCδ inhibitor, attenuated cytoplasmic vacuole formation and significantly increased the survival rate of RGC-5 cells.

Treating RGC-5 cells with EMB significantly increased the caspase-3/7 activity in a dose-dependent manner (Fig. 4C). After an 8-h treatment with 3, 4, 6 or 12 mM EMB, the caspase-3/7 activity in the cells increased by 2.6-, 5.2-, 5.9- and 6.3-fold, respectively, compared with the dimethyl sulfoxide (DMSO)-treated control cells. Rottlerin at a 1 µM concentration significantly reduced the EMB-induced caspase-3/7 activity to nearly the basal level.

**Fig. 5.**
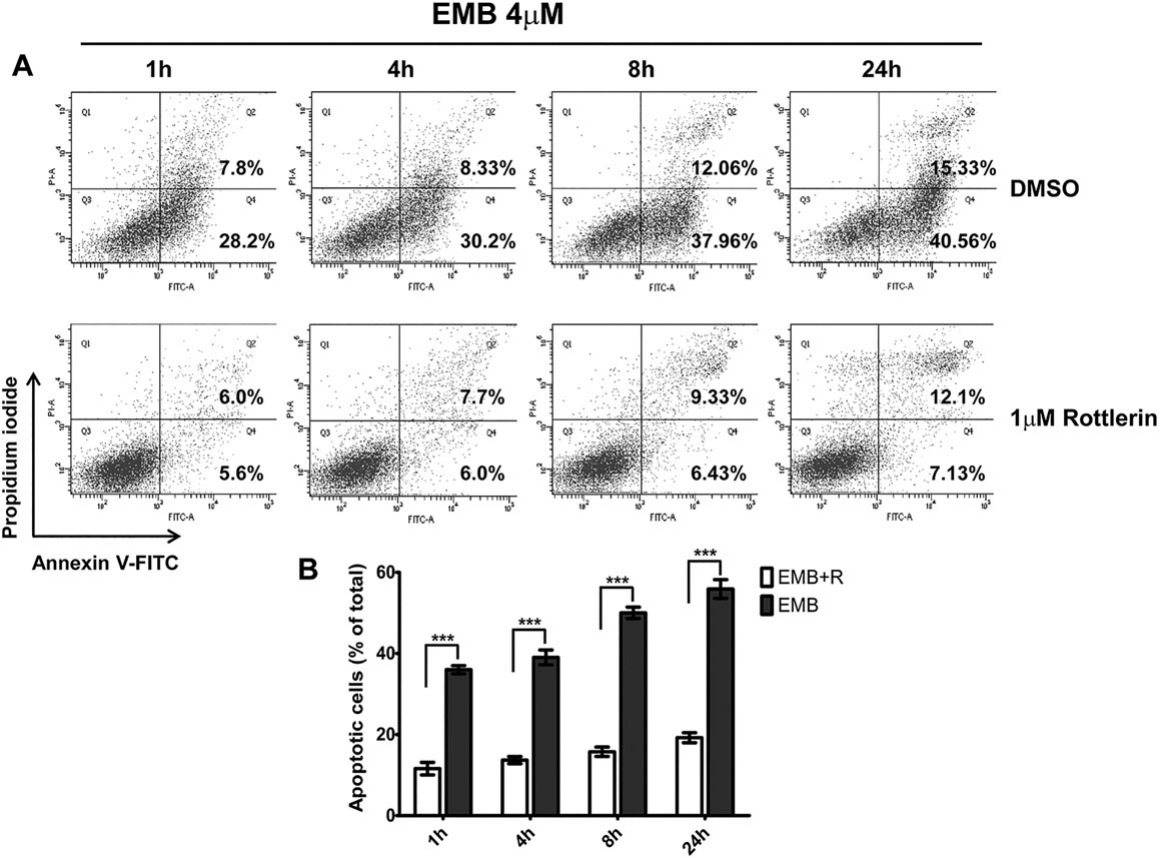
**Annexin V/PI double-staining assay of RGC-5 cells.** (A) Flow cytometric analysis results of EMB-induced apoptosis in RGC-5 cells at different time points are shown. (B) A statistical graph of annexin V-FITC/PI staining is shown. The data averages for each time point were calculated using the results from three independent experiments. The results are expressed as the means±s.e.m. Apoptotic cells included the Annexin V^+^/PI^−^ cells and the Annexin V^+^/PI^+^ cells. ***P<0.001.

To further evaluate cell viability and to determine whether the cell loss observed after EMB treatment was caused by apoptosis, fluorescence-activated cell sorting (FACS) analysis using annexin V and propidium iodide (PI) was performed (Fig. 5A). The FACS analysis confirmed that greater than 90% of non-EMB-treated cells (control) were viable and not labeled with Annexin V or PI (data not shown). Exposure to EMB for 1, 4, 8 and 24 h significantly increased the number of apoptotic cells by 36.0%, 39.03%, 50.03% and 55.9%, respectively. Furthermore, the addition of 1 µM rottlerin to cells treated with EMB for 1, 4, 8 and 24 h significantly reduced the number of apoptotic cells by 11.6%, 13.7%, 15.77% and 19.23%, respectively (all *P*<0.001) (Fig. 5B). These results indicate that the EMB-induced apoptosis of RGC-5 cells is mediated by the activation of PKCδ and the caspase-3 activity.

### Rottlerin reduces EMB-induced phosphorylation of PKCδ and upregulation of autophagic markers in RGC-5 cells

**Fig. 6.**
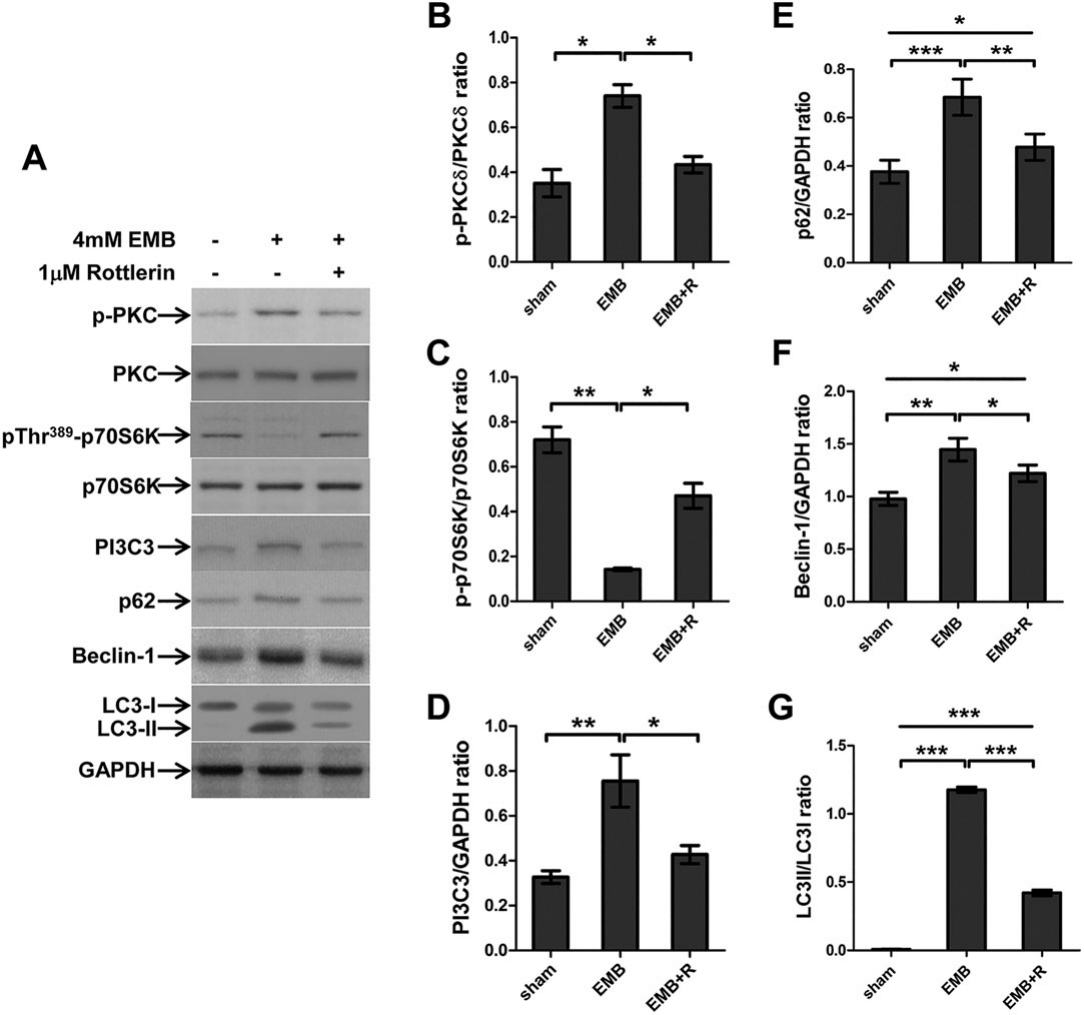
**Rottlerin reduces EMB-induced phosphorylation of PKCδ and upregulation of autophagic markers.** (A) Western blot analysis of the expression levels of PKCδ, phospho-PKCδ, p70S6K, phospho-p70S6K, and the autophagic markers PI3C3, Beclin-1, p62 and LC3 in the total protein extracts from RGC-5 cells of the sham, EMB and EMB+R-treated groups. Representative results from three independent experiments are shown. (B-G) Quantitative analysis of the PKCδ, p70S6K, PI3C3, p62, Beclin-1 and LC3 expression levels normalized to the internal control, GAPDH. The results are expressed as the means±s.e.m. from three independent experiments. *P<0.05, **P<0.01, ***P<0.001.

To gain further insight into the mechanism by which rottlerin attenuates the EMB-induced apoptosis in RGC-5 cells, we examined the levels of PKCδ, p70S6K and autophagic markers by immunoblot analysis (Fig. 6). Rottlerin significantly reduced the EMB-induced phosphorylation of PKCδ (Fig. 6B). The levels of phosphorylated p70S6K (Fig. 6C) were significantly higher in the EMB plus rottlerin (EMB+R)-treated group compared with the EMB-treated group. Rottlerin also significantly suppressed the EMB-induced upregulation of the autophagic markers PI3C3, beclin-1, p62 and LC3II. These data suggest that the EMB-induced PKCδ phosphorylation upregulates autophagy activity through suppression of PI3K/Akt/mTOR/p70S6K signaling.

### EMB induces lysosome dilation and impairs autophagic flux in RGC-5 cells

Treating RGC-5 cells with 4 mM EMB induced cytosolic vacuole formation as soon as 1 h after exposure. To determine whether these vacuoles were derived from specific organelles, we stained RGC-5 cells with a dye specific for lysosomes (LysoTracker; Invitrogen), immunostained the cells with anti-LC3 antibody and then examined the cells via confocal microscopy. In the control cells, the lysosomes were small and very little LC-positive staining was detected (supplementary material Fig. S2A,C); in contrast, in the cells treated with EMB for 4 h, the lysosomes were significantly larger, and more punctate endogenous LC staining was observed (supplementary material Fig. S2B). The PKCδ inhibitor rottlerin inhibited EMB-induced vacuole formation and LC3-positive puncta formation in RGC-5 cells (supplementary material Fig. S2D).

To investigate whether the EMB-induced cytotoxicity was dependent on autophagic flux impairment, we measured autophagic flux in RGC-5 cells using mRFP-GFP tandem fluorescent-tagged LC3 (tfLC3) (Kimura et al., 2007). Because of the different pH stabilities of the green and red fluorescent proteins, the GFP-LC3 loses its fluorescent signal within the acidic lysosomal environment (pH below 5), but the mRFP-LC3 signal persists. Fluorescence images of RGC-5 cells expressing tfLC3 showed yellow puncta (RFP^+^GFP^+^), which are indicative of autophagosomes, and red puncta (RFP^+^GFP^−^), which are indicative of autolysosomes.

**Fig. 7.**
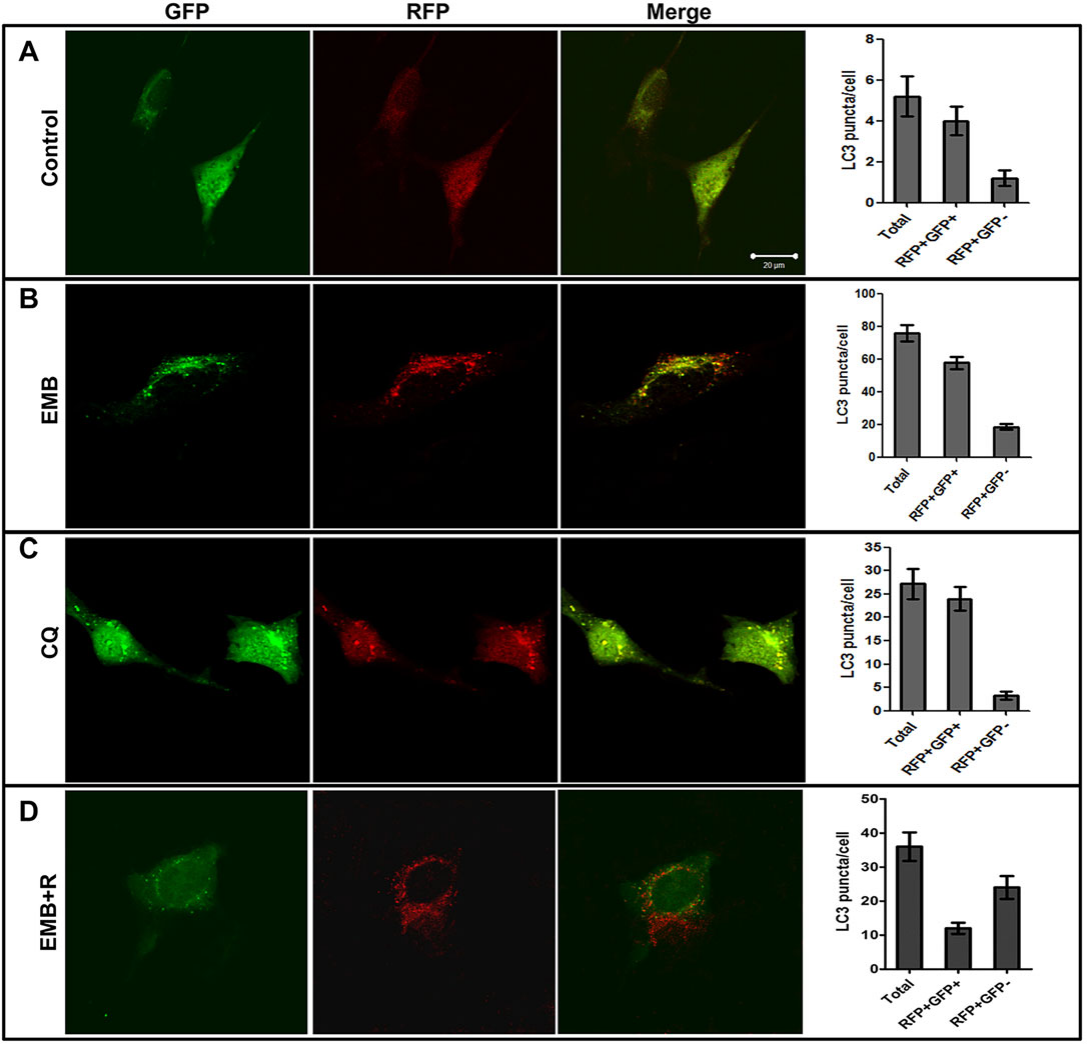
**Monitoring autophagic flux in RGC-5 cells using mRFP-GFP tfLC3.** A comparison of the GFP and RFP tfLC3 signals under different conditions. (A) Representative fluorescent images of and statistical analysis results for RGC-5 cells transiently transfected with tfLC3 without autophagy. (B-D) Representative fluorescent images of and statistical analysis results for RGC-5 cells transiently transfected with tfLC3 and treated with 4 mM EMB (B), 100 µM chloroquine (CQ; C) and 4 mM EMB combined with 1 µM rottlerin (D) for 4 h. Puncta representing the autophagosomes (diameter >75 μm) and autolysosomes per cell were measured using ImageJ software. Scale bar: 20 µm.

Only a small number of LC3 puncta were observed in the control cells (Fig. 7A). When tfLC3 was overexpressed in RGC-5 cells treated with EMB, we detected more autophagosomes (RFP^+^GFP^+^) than autolysosomes (RFP^+^GFP^−^) (Fig. 7B). More than 76% of the LC3 puncta were autophagosomes (RFP^+^GFP^+^) (supplementary material Fig. S3). As expected, when RGC-5 cells were treated with 100 µM chloroquine (CQ), which effectively inhibits the fusion of autophagosomes and lysosomes (Klionsky et al., 2008), approximately 89% of the LC3 puncta were autophagosomes (Fig. 7C). We observed an increase in the number of yellow puncta without a concomitant increase in the number of red LC3 puncta in cells treated with EMB, indicating that the level of autophagosome fusion with lysosomes is lower in the presence of EMB. These results suggest that EMB impairs autophagic flux, which results in autophagosome accumulation in RGC-5 cells. In contrast, in EMB+R-treated RGC-5 cells, the number of red puncta was higher than that of yellow puncta (Fig. 7D). Less than 30% of LC3 puncta are autophagosome. The results indicate that rottlerin attenuates EMB-induced autophagosome accumulation and promotes autophagic flux in RGC-5 cells.

## DISCUSSION

**Fig. 8.**
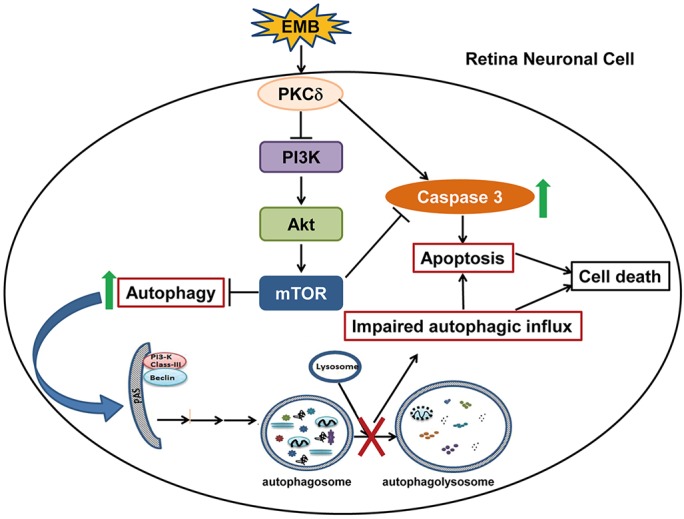
**Hypothetical mechanism for EMB-induced cytotoxicity in retina neuronal cells.** EMB exposure induces PKCδ activation, which in turn suppresses the PI3K/Akt/mTOR pathway, promotes caspase-3/7 activity and is followed by autophagosome accumulation and impaired autophagic influx. The impaired autophagic influx and the increase in caspase-3/7 activation lead to the apoptotic death of retina neuronal cells.

The toxic effect of EMB in RGCs has been confirmed *in vitro* and *in vivo* in rodents (Heng et al., 1999; Yoon et al., 2000). This toxicity is mediated by zinc and lysosomal membrane permeabilization (Chung et al., 2009). Moreover, EMB produces a mitochondrial-coupling defect with a reduction in complex IV activity (Guillet et al., 2010). However, the link between EMB toxicity, RGC degeneration and lysosomal or mitochondrial dysfunction remains to be elucidated. In this study, we demonstrated for the first time that EMB activates PKCδ signaling, mediates caspase-3 activity and inhibits the PI3K/Akt/mTOR pathway, which results in impaired autophagic flux and apoptosis of RGCs. The PKCδ inhibitor rottlerin attenuated EMB-induced cytoplasmic vacuole formation and apoptosis in RGC-5 cells. Based on these findings, we propose a model for understanding the interrelationship between EMB-induced autophagy and apoptosis, which is regulated by PKCδ (Fig. 8). According to this model, EMB induces PKCδ activation and inhibits PI3K/Akt/mTOR signaling, which initially serves to promote autophagy. With sustained EMB treatment, increased PKCδ phosphorylation causes the accumulation of autophagosomes, which fail to fuse with lysosomes, and an increase in caspase-3 activity promotes apoptosis of retina neuronal cells.

PKCδ maintains cellular homeostasis in response to diverse stimuli, including mechanical stress, pro-inflammatory cytokines and oxidative stress (Konishi et al., 2001; Larroque-Cardoso et al., 2013; Qi and Mochly-Rosen, 2008). It has been suggested that PKCδ plays a dual role in regulating autophagy and apoptosis during the early stage of the hypoxic response by promoting JNK1-mediated Bcl-2 phosphorylation and the dissociation of the Bcl-2/Beclin-1 complex, which results in autophagy induction (Chen et al., 2008). Furthermore, prolonged hypoxic stress causes the activation of PKCδ and caspase-3 (Clavijo et al., 2007), which is the major effector in the onset of apoptosis. In this study, we showed that PKCδ activation in EMB-treated retinas is required for the induction of the autophagic process and apoptosis. First, EMB treatment increases the expression level of Beclin-1 and promotes LC3-II formation and the accumulation of GFP-LC3 puncta. Second, EMB treatment induces PKCδ activation and increases caspase-3/7 activity. The sustained activation of the PKCδ and caspase-3 pathways leads to cell death. Furthermore, we demonstrated that rottlerin, a PKCδ inhibitor, attenuates the EMB-induced PKCδ phosphorylation, upregulation of autophagic markers and caspase-3/7 activity, and reduces the apoptotic effect in RGC-5 cells. The previous studies showing that PKCδ-dependent phosphorylation activates caspase-3 (Voss et al., 2005) and that PKCδ suppresses Akt phosphorylation (Clavijo et al., 2007; Murriel et al., 2004), which result in apoptosis induction, support our proposed model. Our findings advance our current understanding of the role that PKCδ plays in the EMB-induced cytotoxicity in the retina and suggest that PKCδ might be involved in the crosstalk between autophagy and apoptosis that regulates the cell fate decision.

mTOR serine/threonine kinase functions as a molecular sensor of the cellular nutrient, energy and redox status, and its activity is inhibited under energy stress. mTOR signaling is a negative regulator of autophagy that ensures that the timing of autophagy induction is stringently controlled (Jung et al., 2010). Furthermore, links between the mTOR and caspase signaling pathways have also been proposed to be involved in regulating cell death (Castedo et al., 2002). In this study, we also investigated the relationship between mTOR signaling and autophagy in EMB-induced cytotoxicity in retina neuronal cells. Our results demonstrate that EMB induces apoptosis in the retina by downregulating the phosphorylation of PI3K, Akt, mTOR and p70S6K and by inhibiting PI3K/AKT/mTOR signaling, which might be responsible for inducing autophagy. The expression levels of the autophagic markers PI3C3, Beclin-1, p62 and LC3-II were higher in EMB-treated retinas, and our immunohistochemical results also indicate that autophagosomes accumulate in EMB-treated retinas. Altogether, our findings suggest that PI3K/AKT/mTOR signaling plays an important role in the mechanism underlying the induction of the autophagy and apoptosis pathways in EMB-treated retinas.

EMB treatment increased the expression levels of Beclin-1 and PI3C3, which form the class III PI3K complex and are involved in the early phase of autophagy. Autophagy is a highly conserved lysosomal degradation process for breaking down and recycling cytoplasmic components and organelles (Glick et al., 2010; Klionsky and Emr, 2000). There are a series of evolutionarily conserved autophagy-related (Atg) proteins, essential for autophagy induction and autophagosome generation, maturation and recycling (Mizushima, 2011). LC3-1 is cleaved and subsequently conjugated with phosphatidylethanolamine to become LC3-II, which is involved in the elongation of autophagosomes. The level of LC3-II correlates with the number of autophagosomes; therefore, the level of conversion of LC3-I to LC3-II can be used as an indicator for autophagic activity (Mizushima et al., 2010). The p62 protein is selectively incorporated into autophagosomes through direct binding to LC3-II and efficiently degraded in the autolysosome. Accordingly, the total p62 expression level is negatively correlated with autophagic flux (Mizushima et al., 2010). The western blot analysis revealed that the expression levels of LC3-II and p62 were significantly higher in EMB-treated retinas, which indicates that autophagic degradation and the fusion between autophagosomes and lysosomes have been inhibited. Therefore, it seems that the induction of autophagy by EMB is a secondary effect of the impaired autophagic flux. Similar conclusions were reached when autophagic flux was evaluated using the tfLC3 assay (Kimura et al., 2007). Consistent with the immunoblotting analysis results, RGC-5 cells treated with EMB demonstrated a decrease in autophagic flux, as shown by the increase in the number of yellow puncta (autophagosomes) without the concomitant increase in the number of red LC3 puncta (autolysosomes) per cell. These results imply that EMB treatment might impair the fusion of autophagosomes and lysosomes and might thus inhibit autophagic flux. Furthermore, we also demonstrated that treating RGC-5 cells with EMB leads to lysosome dilation. Our results are consistent with the results from previous studies suggesting that the site of EMB toxic activity is the lysosome (Chung et al., 2009). These findings indicate that dysregulation in the autophagy-dependent protein degradation in the lysosomes might contribute to EMB-induced optic neuroretinopathy.

RGC-5 cells have been widely used to investigate the cellular and molecular mechanism of neuronal cell death in the retina. However, the RGC-5 cells are now reported to express photoreceptor marker and share certain features with 661W cells (Krishnamoorthy et al., 2013), an SV-40T-antigen-transformed mouse photoreceptor cell line (Tan et al., 2004). RGC-5 cells do not seem to be a suitable model for RGCs. This is a potential limitation of our study. Furthermore, abnormal electro-oculogram (EOG) (Yen et al., 2000) and decreased amplitude or delayed implicit time in multifocal electroretinogram (ERG) (Behbehani et al., 2005; Kardon et al., 2006; Lai et al., 2009) have been reported in some patients taking EMB. These clinical studies suggest the visual dysfunction might be attributable to toxicity of the retina rather than optic nerve. The toxic effects of EMB on retinal cells were also highlighted in previous studies (Chung et al., 2009; Liu et al., 2008; Tsai et al., 2008a; Vistamehr et al., 2007). We also observed the LC3-stained punctate granules not only in the RGC but also in the inner segments of the photoreceptor and RPE cells (Fig. 2I,J) in the EMB-treated retina. This expression pattern is consistent with the autophagosome formation induced by EMB. The accumulated evidence indicates that the cytotoxic effect of EMB on the retinal cells might be associated with autophagy dysfunction. However, isolating and culturing primary retinal neuronal cells, such as RGCs or photoreceptors, is technically difficult and results in a limited number of cells that only survive for a few days (Grozdanov et al., 2010). Therefore, the goal of this study was to use the RGC-5 cell line to explore the molecular mechanism of EMB cytotoxicity in the retinal neuronal cells with the expectation that the *in vitro* data set obtained from RGC-5 cells can be verified in primary retinal neuronal cells in a future study. Furthermore, an *in vitro* cell line has high homogeneity and is easy to handle and manipulate, especially in the assay we used to monitor autophagic flux by tandem fluorescent-tagged LC3 for the analysis of autophagy in the neuronal cells. Most importantly, a stable immortal cell line better representing RGC characteristics should be developed in the near future.

In summary, we demonstrated that EMB causes impaired autophagic flux and apoptosis through the activation of PKCδ and the suppression of PI3K/Akt/mTOR signaling in rat retinas. Moreover, EMB-induced phosphorylation of PKCδ, upregulation of autophagic markers, and activation of caspase-3 activity and apoptosis in RGC-5 cells can be attenuated with the PKCδ inhibitor rottlerin. These results further expand our understanding of the mechanism underlying the cytotoxic effects of EMB in the retina and provide new insights that might promote the development of protective strategies against EMB-induced optic neuroretinopathy.

## MATERIALS AND METHODS

### Animal experiment for toxic effects of EMB

Thirty adult male Wistar rats weighing 150-180 g were treated with EMB (35 mg/kg body weight per day) by daily IP injection for 10 consecutive days. The EMB dose was increased by approximately 40% over the recommended dose (25 mg/kg body weight per day) to ensure that the ocular side-effects were induced by EMB after 10 days of administration. Twenty rats were treated with PBS and served as controls. All of the animal experiments were performed in accordance with the ARVO statement for the use of Animals in Ophthalmic and Vision Research and were approved by the IACUC at Tzu Chi General Hospital.

### Flash visual-evoked potentials (FVEPs)

For the functional evaluation of the optic nerve, FVEPs were recorded 10 days after the intraperitoneal injection of EMB or PBS in rats. An isolated silver plate electrode was placed extradurally through a 2-mm diameter craniotomy over the visual cortex using the stereotactic coordinates (bregma −8 mm, lateral 3 mm) and a modified method described by Ohlsson et al. (2004). We used a visual electrodiagnostic system (UTAS-E3000, LKC Technologies, Gaithersburg, MD) to measure FVEPs (Tsai et al., 2008b). Briefly, the recording electrodes in the occipital area and a reference electrode in the frontal area were connected separately with silver wires while the rat was under general anesthesia. An ear clip was placed firmly on the ear lobe. The settings were background illumination off, a flash intensity of Ganzfeld 0 db, single flash with flash rate on 1.9 Hz, the test average at 80 sweeps, the threshold for rejecting artifacts at 50 mV and a sample rate of 2000 Hz. We compared the latency of the first positive wave (P1) of the FVEP between the sham and EMB-treated groups (*n*=6 in each group).

### Cell culture and viability assays

The RGC-5 cell line was kindly provided by Dr Neeraj Agarwal (National Eye Institute, Bethesda, MD). RGC-5 cells were maintained in low-glucose Dulbecco's modified Eagle's medium containing 10% fetal bovine serum (FBS) in a humidified atmosphere with 5% CO_2_ at 37°C. Cell viability, evaluated as mitochondrial activity, was determined by measuring the dehydrogenase activity retained in the cells using the MTT (Sigma-Aldrich) assay. The assay is based on the ability of living cells to reduce MTT into insoluble formazan. Briefly, cells (1500 cell per well) were seeded onto 96-well plates and treated with 0 to 12 mM EMB for 24 h. After 24 h, the cells were incubated in 0.5 mg/ml MTT (100 ml per well) for 2 h in a humidified 5% CO_2_ incubator at 37°C. The medium was then removed, and 100 ml of DMSO (Sigma-Aldrich) was added to solubilize the formazan product. The absorbance was read at 540/690 nm using an enzyme-linked immunosorbent assay (ELISA) reader (Labsystems Multiskan, Helsinki, Finland). Data were expressed as cell survival percentages compared with the control cultures (maintained in 10% FBS), which were set to 100%.

### Assays of caspase-3/7 activity

RGC-5 cells were cultured, and the caspase-3/7 activity was measured using an assay kit (Apo-ONE Homogeneous Caspase-3/7 activity; Promega, Madison, WI) according to the manufacturer's protocol. Briefly, the assay reagent (100 µl) was directly added to each well at room temperature. The plates were shaken at 500 rpm for 30 s, and caspase-3/7 activation was determined by measuring the fluorescence intensity (excitation at 480 nm, emission at 520 nm) using a fluorescence plate reader. The intensity was expressed as the fold change compared with the control. At least three independent replicates were run for each set of experiments to confirm the consistency of our findings.

### Flow cytometry for apoptosis detection

RGC-5 cells were seeded onto 24-well plates and treated with DMSO (control), 4 mM EMB or 4 mM EMB/rottlerin and incubated for 1, 4, 8 or 24 h. Apoptosis was measured by flow cytometry using the fluorescein isothiocyanate (FITC) Annexin V/Dead Cell Apoptosis Kit (Invitrogen) according to the manufacturer's protocol. Briefly, the cells were dissociated with trypsin, fixed in 70% ethanol at −20°C and incubated in dichloro-fluorescein (DCF, 5 mM, Invitrogen) for 30 min at 37°C to measure the intracellular reactive oxygen species (ROS) production in the viable cells. 3,3′-dihexyloxacarbocyanine iodide [DiOC_6_(3), 50 nM 15 min; Invitrogen] was used to determine the mitochondrial membrane potential, and PI (1 mg/ml, Sigma) was used to determine the cell cycle distribution. After staining, the cellular DNA content was evaluated by FACS flow cytometry using Cell Quest software (Becton Dickinson and Company, USA). The proportion of cells in each cell cycle phase was determined using the Windows multiple document interface for flow cytometry (WinMDI) software.

### Immunoblot analysis

Protein extracts from EMB-treated or sham group rat retinas were prepared using NP-40 lysis buffer (50 mM Tris-HCl pH 7.6, 150 mM NaCl, 1% NP-40 and 1 mM EDTA). After washing with serum-free medium, RGC-5 cells treated with DMSO (control), 4 mM EMB or 4 mM EMB/rottlerin for 24 h were suspended in NP-40 lysis buffer and centrifuged. The protein concentrations were determined using the bicinchoninic acid (BCA) protein assay kit (Pierce). The same amount of protein from each sample was separated by 10% sodium dodecyl sulfate/polyacrylamide gel electrophoresis (SDS/PAGE) and then transferred to polyvinylidene difluoride (PVDF) membranes. Membranes were blocked in 5% dry milk and were then probed with different primary antibodies [anti-PI3C3, anti-phospho-PKCδ, anti-Akt, anti-phospho-Akt, anti-mTOR, anti-phospho-mTOR, anti-p70S6K, anti-phospho-p70S6K, anti-caspase, anti-cleaved caspase-3 (Cell Signaling), anti-Beclin-1, anti-LC3 (Abcam), anti-p62 and anti-PKCδ (Santa Cruz Biotechnology)] overnight at 4°C. After washing, the blots were incubated in the appropriate anti-horseradish-peroxidase-conjugated secondary antibody (1:10,000; Bio-Rad) at room temperature for 1 h. The proteins on the membranes were detected using an enhanced chemiluminescence (ECL) system (Amersham Biosciences). The blots were also probed with an antibody for glyceraldehyde-3-phosphate dehydrogenase (GAPDH) as an internal loading control. Densitometric analysis was conducted using ImageJ software. Each experiment was repeated three times with independent retinal samples from different animals.

### Immunohistochemistry

To further evaluate the LC3 expression patterns in the retinas from the EMB-treated and sham groups, we used a fluorescent immunohistochemical staining method as previously described (Huang et al., 2010). Briefly, the eyes were enucleated, the cornea was removed and the eyes were immediately immersed in 4% (w/v) paraformaldehyde (PFA) in 0.1 M PBS for 2 h at room temperature. The eyes were rinsed in PBS (pH 7.4), cryoprotected in a 30% sucrose-PBS solution overnight at 4°C and then embedded in OCT (ornithine carbamyl transferase) (Tissue-Tek). Sections (7 µm) were cut through the optic nerve of the retina using a cryostat. The collected sections were washed in 0.1 M PBS, blocked in blocking buffer [1% bovine serum albumin (BSA), 1% NGS, 1% Triton X-100 in 1× PBS] and incubated in an anti-rabbit LC3 polyclonal antibody (PAb, 1:2000; Abcam) overnight at 4°C. To visualize the bound primary antibody, sections were incubated in secondary antibody conjugated to Alexa Fluor 488 (1:500; Invitrogen) and TO-PRO-3 (1:2500; Invitrogen), a nuclear stain, for 1 h at room temperature. The sections were visualized and photographed using a Zeiss confocal laser-scanning microscope (Carl Zeiss).

### Plasmids and cell transfection

The mRFP-eGFP-LC3 construct created by T. Yoshimori (Kimura et al., 2007) (ptfLC3; Addgene plasmid 21074) was obtained from Addgene. Cells were pooled, seeded in chamber slides and cultured for 24 h before treatment. To analyze the autophagic flux, RGC-5 cells were transfected with ptfLC3-expressing plasmid using XtremeGENE 9 (1/2 ratio) according to the manufacturer's instructions (Roche Diagnostics) for 48 h and then treated with 4 mM EMB, 4 mM EMB and 1 µM rottlerin, 100 µM CQ or DMSO for 4 h. Cells were washed in PBS and fixed in freshly prepared 4% PFA. After three washes with PBS, the cells were mounted. Autophagic flux was determined by evaluating patterns of GFP and RFP puncta under a Zeiss confocal laser-scanning microscope and quantifying the LC3 puncta (puncta/cell were counted) using ImageJ software. Results were obtained from three independent experiments with at least 100 cells analyzed.

### Immunocytochemistry

RGC-5 cells were treated with 4 mM EMB or DMSO for 4 h and then stained with 75 nM LysoTracker Red DND-99 (Invitrogen) in DMEM for 30 min in a humidified CO_2_ incubator. Next, the cells were fixed with 4% (v/v) paraformaldehyde for 15 min at room temperature and permeabilized with 0.2% (v/v) Triton X-100 for 15 min. After blocking with 2% (w/v) BSA, the fixed cells were incubated in anti-LC3 (1:400; Abcam) antibody for 1 h at room temperature. The stained cells were washed and then incubated in a fluorescence-conjugated secondary antibody (Alexa-Fluor-568–goat anti-rabbit IgG, 1:500; Invitrogen) and TO-PRO-3 (Invitrogen) for 1 h at room temperature. For negative controls, cultured cells were incubated in secondary antibody only.

### Statistical analysis

Statistical significance was determined using a two-tailed Student's *t*-test and a one-way ANOVA followed by Bonferroni's multiple comparison test. Data are presented as the means±s.e.m. In all cases, a *P*<0.05 was considered statistically significant.

## Supplementary Material

Supplementary Material

## References

[DMM019737C1] Alvarez K. L. and Krop L. C. (1993). Ethambutol-induced ocular toxicity revisited. *Ann. Pharmacother.* 27, 102-103. 8431610 10.1177/106002809302700126

[DMM019737C2] Basu A., Mohanty S. and Sun B. (2001). Differential sensitivity of breast cancer cells to tumor necrosis factor-alpha: involvement of protein kinase C. *Biochem. Biophys. Res. Commun.* 280, 883-891. 10.1006/bbrc.2000.4209 11162606

[DMM019737C3] Behbehani R. S., Affel E. L., Sergott R. C. and Savino P. J. (2005). Multifocal ERG in ethambutol associated visual loss. *Br. J. Ophthalmol.* 89, 976-982. 10.1136/bjo.2004.065656 16024847 PMC1772772

[DMM019737C4] Boya P., Gonzalez-Polo R.-A., Casares N., Perfettini J.-L., Dessen P., Larochette N., Metivier D., Meley D., Souquere S., Yoshimori T. et al. (2005). Inhibition of macroautophagy triggers apoptosis. *Mol. Cell. Biol.* 25, 1025-1040. 10.1128/MCB.25.3.1025-1040.2005 15657430 PMC543994

[DMM019737C5] Campbell I. A. and Elmes P. C. (1975). Ethambutol and the eye; zinc and copper. *Lancet* 306, 711 10.1016/S0140-6736(75)90812-0 52087

[DMM019737C6] Castedo M., Ferri K. F. and Kroemer G. (2002). Mammalian target of rapamycin (mTOR): pro- and anti-apoptotic. *Cell Death Differ.* 9, 99-100. 10.1038/sj.cdd.4400978 11840159

[DMM019737C7] Chai S. J. and Foroozan R. (2007). Decreased retinal nerve fibre layer thickness detected by optical coherence tomography in patients with ethambutol-induced optic neuropathy. *Br. J. Ophthalmol.* 91, 895-897. 10.1136/bjo.2006.113118 17215265 PMC1955652

[DMM019737C8] Chen J.-L., Lin H. H., Kim K.-J., Lin A., Forman H. J. and Ann D. K. (2008). Novel roles for protein kinase Cdelta-dependent signaling pathways in acute hypoxic stress-induced autophagy. *J. Biol. Chem.* 283, 34432-34444. 10.1074/jbc.M804239200 18836180 PMC2590682

[DMM019737C9] Chen J.-L., Lin H. H., Kim K.-J., Lin A., Ou J.-H. J. and Ann D. K. (2009). PKC delta signaling: a dual role in regulating hypoxic stress-induced autophagy and apoptosis. *Autophagy* 5, 244-246. 10.4161/auto.5.2.7549 19098423 PMC2743529

[DMM019737C10] Chung Y. M., Yeh T. S., Sheu S. J. and Liu J. H. (1989). Macular subretinal neovascularization in choroidal tuberculosis. *Ann. Ophthalmol.* 21, 225-229. 2475047

[DMM019737C11] Chung H., Yoon Y. H., Hwang J. J., Cho K. S., Koh J. Y. and Kim J.-G. (2009). Ethambutol-induced toxicity is mediated by zinc and lysosomal membrane permeabilization in cultured retinal cells. *Toxicol. Appl. Pharmacol.* 235, 163-170. 10.1016/j.taap.2008.11.006 19063910

[DMM019737C12] Clavijo C., Chen J.-L., Kim K.-J., Reyland M. E. and Ann D. K. (2007). Protein kinase Cdelta-dependent and -independent signaling in genotoxic response to treatment of desferroxamine, a hypoxia-mimetic agent. *Am. J. Physiol. Cell Physiol.* 292, C2150-C2160. 10.1152/ajpcell.00425.2006 17563398

[DMM019737C13] DeVita E. G., Miao M. and Sadun A. A. (1987). Optic neuropathy in ethambutol-treated renal tuberculosis. *J. Clin. Neuroophthalmol.* 7, 77-86. 2956288

[DMM019737C14] Figueroa R., Weiss H., Smith J. C. Jr, Hackley B. M., McBean L. D., Swassing C. R. and Halsted J. A. (1971). Effect of ethambutol on the ocular zinc concentration in dogs. *Am. Rev. Respir. Dis.* 104, 592-594. 5094059 10.1164/arrd.1971.104.4.592

[DMM019737C15] Ghayur T., Hugunin M., Talanian R. V., Ratnofsky S., Quinlan C., Emoto Y., Pandey P., Datta R., Huang Y., Kharbanda S. et al. (1996). Proteolytic activation of protein kinase C delta by an ICE/CED 3-like protease induces characteristics of apoptosis. *J. Exp. Med.* 184, 2399-2404. 10.1084/jem.184.6.2399 8976194 PMC2196396

[DMM019737C16] Ginet V., Puyal J., Clarke P. G. H. and Truttmann A. C. (2009). Enhancement of autophagic flux after neonatal cerebral hypoxia-ischemia and its region-specific relationship to apoptotic mechanisms. *Am. J. Pathol.* 175, 1962-1974. 10.2353/ajpath.2009.090463 19815706 PMC2774060

[DMM019737C17] Glick D., Barth S. and Macleod K. F. (2010). Autophagy: cellular and molecular mechanisms. *J. Pathol.* 221, 3-12. 10.1002/path.2697 20225336 PMC2990190

[DMM019737C18] Grozdanov V., Muller A., Sengottuvel V., Leibinger M. and Fischer D. (2010). A method for preparing primary retinal cell cultures for evaluating the neuroprotective and neuritogenic effect of factors on axotomized mature CNS neurons. *Curr. Protoc. Neurosci.* Chapter 3, Unit 3.22 10.1002/0471142301.ns0322s53 20938922

[DMM019737C19] Guillet V., Chevrollier A., Cassereau J., Letournel F., Gueguen N., Richard L., Desquiret V., Verny C., Procaccio V., Amati-Bonneau P. et al. (2010). Ethambutol-induced optic neuropathy linked to OPA1 mutation and mitochondrial toxicity. *Mitochondrion* 10, 115-124. 10.1016/j.mito.2009.11.004 19900585

[DMM019737C20] Heng J. E., Vorwerk C. K., Lessell E., Zurakowski D., Levin L. A. and Dreyer E. B. (1999). Ethambutol is toxic to retinal ganglion cells via an excitotoxic pathway. *Invest. Ophthalmol. Vis. Sci.* 40, 190-196. 9888443

[DMM019737C21] Huang S.-P., Brown B. M. and Craft C. M. (2010). Visual Arrestin 1 acts as a modulator for N-ethylmaleimide-sensitive factor in the photoreceptor synapse. *J. Neurosci.* 30, 9381-9391. 10.1523/JNEUROSCI.1207-10.2010 20631167 PMC2920134

[DMM019737C22] Jung C. H., Ro S.-H., Cao J., Otto N. M. and Kim D.-H. (2010). mTOR regulation of autophagy. *FEBS Lett.* 584, 1287-1295. 10.1016/j.febslet.2010.01.017 20083114 PMC2846630

[DMM019737C23] Kardon R. H., Morrisey M. C. and Lee A. G. (2006). Abnormal multifocal electroretinogram (mfERG) in ethambutol toxicity. *Semin. Ophthalmol.* 21, 215-222. 10.1080/08820530600987454 17182409

[DMM019737C24] Kimura S., Noda T. and Yoshimori T. (2007). Dissection of the autophagosome maturation process by a novel reporter protein, tandem fluorescent-tagged LC3. *Autophagy* 3, 452-460. 10.4161/auto.4451 17534139

[DMM019737C25] Klionsky D. J. and Emr S. D. (2000). Autophagy as a regulated pathway of cellular degradation. *Science* 290, 1717-1721. 10.1126/science.290.5497.1717 11099404 PMC2732363

[DMM019737C26] Klionsky D. J. Abeliovich H. Agostinis P. Agrawal D. K. Aliev G. Askew D. S. Baba M. Baehrecke E. H. Bahr B. A. Ballabio A. et al. (2008). Guidelines for the use and interpretation of assays for monitoring autophagy in higher eukaryotes. *Autophagy* 4, 151-175. 10.4161/auto.5338 18188003 PMC2654259

[DMM019737C27] Koike M., Shibata M., Tadakoshi M., Gotoh K., Komatsu M., Waguri S., Kawahara N., Kuida K., Nagata S., Kominami E. et al. (2008). Inhibition of autophagy prevents hippocampal pyramidal neuron death after hypoxic-ischemic injury. *Am. J. Pathol.* 172, 454-469. 10.2353/ajpath.2008.070876 18187572 PMC2312361

[DMM019737C28] Konishi H., Yamauchi E., Taniguchi H., Yamamoto T., Matsuzaki H., Takemura Y., Ohmae K., Kikkawa U. and Nishizuka Y. (2001). Phosphorylation sites of protein kinase C delta in H2O2-treated cells and its activation by tyrosine kinase in vitro. *Proc. Natl. Acad. Sci. USA* 98, 6587-6592. 10.1073/pnas.111158798 11381116 PMC34397

[DMM019737C29] Krishnamoorthy R. R., Clark A. F., Daudt D., Vishwanatha J. K. and Yorio T. (2013). A forensic path to RGC-5 cell line identification: lessons learned. *Invest. Ophthalmol. Vis. Sci.* 54, 5712-5719. 10.1167/iovs.13-12085 23975727

[DMM019737C30] Lai T. Y. Y., Ngai J. W. S., Lai R. Y. K. and Lam D. S. C. (2009). Multifocal electroretinography changes in patients on ethambutol therapy. *Eye* 23, 1707-1713. 10.1038/eye.2008.361 19675572

[DMM019737C31] Larroque-Cardoso P., Swiader A., Ingueneau C., Nègre-Salvayre A., Elbaz M., Reyland M. E., Salvayre R. and Vindis C. (2013). Role of protein kinase C delta in ER stress and apoptosis induced by oxidized LDL in human vascular smooth muscle cells. *Cell Death Dis.* 4, e520 10.1038/cddis.2013.47 23449456 PMC3734829

[DMM019737C32] Lee E. J., Kim S.-J., Choung H. K., Kim J. H. and Yu Y. S. (2008). Incidence and clinical features of ethambutol-induced optic neuropathy in Korea. *J. Neuroophthalmol.* 28, 269-277. 10.1097/WNO.0b013e31818e3c6b 19145123

[DMM019737C33] Liu Y., Dinkin M. J., Loewenstein J. I., Rizzo J. F. III and Cestari D. M. (2008). Multifocal electroretinographic abnormalities in ethambutol-induced visual loss. *J. Neuroophthalmol.* 28, 278-282. 10.1097/WNO.0b013e31818e3ece 19145124

[DMM019737C34] Menon V., Jain D., Saxena R. and Sood R. (2009). Prospective evaluation of visual function for early detection of ethambutol toxicity. *Br. J. Ophthalmol.* 93, 1251-1254. 10.1136/bjo.2008.148502 19525243

[DMM019737C35] Mizushima N. (2004). Methods for monitoring autophagy. *Int. J. Biochem. Cell Biol.* 36, 2491-2502. 10.1016/j.biocel.2004.02.005 15325587

[DMM019737C36] Mizushima N. (2011). Autophagy in protein and organelle turnover. *Cold Spring Harb. Symp. Quant. Biol.* 76, 397-402. 10.1101/sqb.2011.76.011023 21813637

[DMM019737C37] Mizushima N., Yoshimori T. and Levine B. (2010). Methods in mammalian autophagy research. *Cell* 140, 313-326. 10.1016/j.cell.2010.01.028 20144757 PMC2852113

[DMM019737C38] Murriel C. L., Churchill E., Inagaki K., Szweda L. I. and Mochly-Rosen D. (2004). Protein kinase Cdelta activation induces apoptosis in response to cardiac ischemia and reperfusion damage: a mechanism involving BAD and the mitochondria. *J. Biol. Chem.* 279, 47985-47991. 10.1074/jbc.M405071200 15339931

[DMM019737C39] Nixon R. A. (2006). Autophagy in neurodegenerative disease: friend, foe or turncoat? *Trends Neurosci.* 29, 528-535. 10.1016/j.tins.2006.07.003 16859759

[DMM019737C40] Ohlsson M., Mattsson P. and Svensson M. (2004). A temporal study of axonal degeneration and glial scar formation following a standardized crush injury of the optic nerve in the adult rat. *Restor. Neurol. Neurosci.* 22, 1-10. 15096689

[DMM019737C41] Ozpolat B., Akar U., Mehta K. and Lopez-Berestein G. (2007). PKC delta and tissue transglutaminase are novel inhibitors of autophagy in pancreatic cancer cells. *Autophagy* 3, 480-483. 10.4161/auto.4349 17507797

[DMM019737C42] Pattingre S., Espert L., Biard-Piechaczyk M. and Codogno P. (2008). Regulation of macroautophagy by mTOR and Beclin 1 complexes. *Biochimie* 90, 313-323. 10.1016/j.biochi.2007.08.014 17928127

[DMM019737C43] Pattingre S., Bauvy C., Carpentier S., Levade T., Levine B. and Codogno P. (2009). Role of JNK1-dependent Bcl-2 phosphorylation in ceramide-induced macroautophagy. *J. Biol. Chem.* 284, 2719-2728. 10.1074/jbc.M805920200 19029119 PMC2631952

[DMM019737C44] Pickford F., Masliah E., Britschgi M., Lucin K., Narasimhan R., Jaeger P. A., Small S., Spencer B., Rockenstein E., Levine B. et al. (2008). The autophagy-related protein beclin 1 shows reduced expression in early Alzheimer disease and regulates amyloid beta accumulation in mice. *J. Clin. Invest.* 118, 2190-2199. 18497889 10.1172/JCI33585 PMC2391284

[DMM019737C45] Qi X. and Mochly-Rosen D. (2008). The PKCdelta -Abl complex communicates ER stress to the mitochondria - an essential step in subsequent apoptosis. *J. Cell Sci.* 121, 804-813. 10.1242/jcs.024653 18285444

[DMM019737C46] Ravikumar B., Sarkar S., Davies J. E., Futter M., Garcia-Arencibia M., Green-Thompson Z. W., Jimenez-Sanchez M., Korolchuk V. I., Lichtenberg M., Luo S. et al. (2010). Regulation of mammalian autophagy in physiology and pathophysiology. *Physiol. Rev.* 90, 1383-1435. 10.1152/physrev.00030.2009 20959619

[DMM019737C47] Renna M., Jimenez-Sanchez M., Sarkar S. and Rubinsztein D. C. (2010). Chemical inducers of autophagy that enhance the clearance of mutant proteins in neurodegenerative diseases. *J. Biol. Chem.* 285, 11061-11067. 10.1074/jbc.R109.072181 20147746 PMC2856980

[DMM019737C48] Sivakumaran P., Harrison A. C., Marschner J. and Martin P. (1998). Ocular toxicity from ethambutol: a review of four cases and recommended precautions. *N. Z. Med. J.* 111, 428-430. 9861923

[DMM019737C49] Tan E., Ding X.-Q., Saadi A., Agarwal N., Naash M. I. and Al-Ubaidi M. R. (2004). Expression of cone-photoreceptor-specific antigens in a cell line derived from retinal tumors in transgenic mice. *Invest. Ophthalmol. Vis. Sci.* 45, 764-768. 10.1167/iovs.03-1114 14985288 PMC2937568

[DMM019737C50] Tsai R. K., Chang C. H., Hseu C. M., Chang S. M., Wu J.-R., Wang H.-Z., Wu W. C. and Wu W. S. (2008a). Ethambutol induces PKC-dependent cytotoxic and antiproliferative effects on human retinal pigment cells. *Exp. Eye Res.* 87, 594-603. 10.1016/j.exer.2008.09.013 18948097

[DMM019737C51] Tsai R. K., Chang C. H. and Wang H. Z. (2008b). Neuroprotective effects of recombinant human granulocyte colony-stimulating factor (G-CSF) in neurodegeneration after optic nerve crush in rats. *Exp. Eye Res.* 87, 242-250. 10.1016/j.exer.2008.06.004 18602391

[DMM019737C52] Tsai R. K., He M. S., Chen Z. Y., Wu W. C. and Wu W. S. (2011). PKCdelta-dependent signaling mediates ethambutol-induced toxic effects on human retinal pigment cells. *Mol. Vis.* 17, 1564-1576. 21738386 PMC3123160

[DMM019737C53] Vistamehr S., Walsh T. J. and Adelman R. A. (2007). Ethambutol neuroretinopathy. *Semin. Ophthalmol.* 22, 141-146. 10.1080/08820530701457134 17763233

[DMM019737C54] Voss O. H., Kim S., Wewers M. D. and Doseff A. I. (2005). Regulation of monocyte apoptosis by the protein kinase Cdelta-dependent phosphorylation of caspase-3. *J. Biol. Chem.* 280, 17371-17379. 10.1074/jbc.M412449200 15716280

[DMM019737C55] Wong E. and Cuervo A. M. (2010). Integration of clearance mechanisms: the proteasome and autophagy. *Cold Spring Harb. Perspect. Biol.* 2, a006734 10.1101/cshperspect.a006734 21068151 PMC2982176

[DMM019737C56] Wooten M. W. (1999). Function for NF-kB in neuronal survival: regulation by atypical protein kinase C. *J. Neurosci. Res.* 58, 607-611. 10.1002/(SICI)1097-4547(19991201)58:5<607::AID-JNR1>3.0.CO;2-M 10561688

[DMM019737C57] Woung L.-C., Jou J.-R. and Liaw S.-L. (1995). Visual function in recovered ethambutol optic neuropathy. *J. Ocul. Pharmacol. Ther.* 11, 411-419. 10.1089/jop.1995.11.411 8590273

[DMM019737C58] Yang Z. and Klionsky D. J. (2010). Mammalian autophagy: core molecular machinery and signaling regulation. *Curr. Opin. Cell Biol.* 22, 124-131. 10.1016/j.ceb.2009.11.014 20034776 PMC2854249

[DMM019737C59] Yen M. Y., Wang A. G., Chiang S. C. and Liu J. H. (2000). Ethambutol retinal toxicity: an electrophysiologic study. *J. Formos. Med. Assoc.* 99, 630-634. 10969506

[DMM019737C60] Yiannikas C., Walsh J. C. and McLeod J. G. (1983). Visual evoked potentials in the detection of subclinical optic toxic effects secondary to ethambutol. *Arch. Neurol.* 40, 645-648. 10.1001/archneur.1983.04050090081014 6615272

[DMM019737C61] Yoon Y. H., Jung K. H., Sadun A. A., Shin H.-C. and Koh J.-Y. (2000). Ethambutol-induced vacuolar changes and neuronal loss in rat retinal cell culture: mediation by endogenous zinc. *Toxicol. Appl. Pharmacol.* 162, 107-114. 10.1006/taap.1999.8846 10637134

[DMM019737C62] Zoumalan C. I., Agarwal M. and Sadun A. A. (2005). Optical coherence tomography can measure axonal loss in patients with ethambutol-induced optic neuropathy. *Graefes Arch. Clin. Exp. Ophthalmol.* 243, 410-416. 10.1007/s00417-004-1053-1 15565293

