# Supplementary material for: Ethambutol induces impaired autophagic flux and apoptosis in the rat retina
Source: Dis Model Mech. 2015 Aug 1;8(8):977–87. doi: 10.1242/dmm.019737 (PMC4527287; doi:10.1242/dmm.019737)
Supplement: Supplementary Material [file supp_8_8_977__index.html]

Supplementary Material 

# Ethambutol induces impaired autophagic flux and apoptosis in the rat retina

## DMM019737 Supplementary Material

- Supplementary Material
