# Supplementary material for: Ethambutol induces impaired autophagic flux and apoptosis in the rat retina
Source: Dis Model Mech. 2015 Aug 1;8(8):977–87. doi: 10.1242/dmm.019737 (PMC4527287; doi:10.1242/dmm.019737)
Supplement: Supplementary Material [file supp_019737_DMM019737supp.pdf]

Supplementary Figures

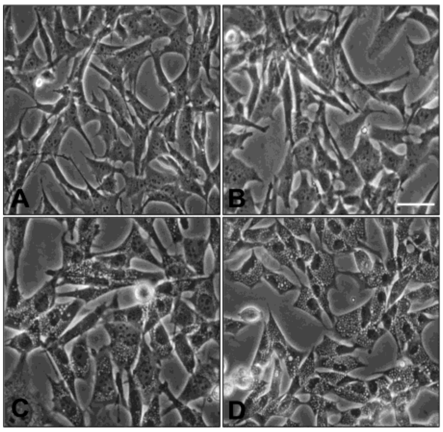

Supplementary Figure 1. EMB induces cytoplasmic vacuole formation in RGC-5 cells. RGCs were treated with (A) DMSO, (B) 4 mM EMB, (C) 6 mM EMB and (D) 12 mM EMB for 4 h.

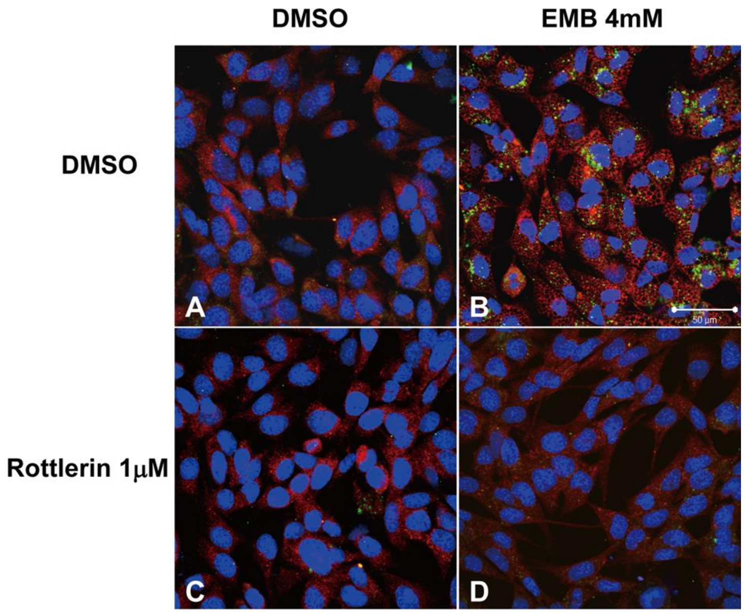

Supplementary Figure 2. EMB treatment induces lysosomal dilation in RGC-5 cells. Confocal microscopy images of LysoTracker (red) and immunofluorescence staining of endogenous LC3 (green) in RGC-5 cells after treating with DMSO (A), 4 mM EMB (B), DMSO and 1 μM Rottlerin (C) or 4 mM EMB and 1 μM rottlerin (D) for 4 h. The number of cytoplasmic vacuoles that formed after EMB treatment corresponded closely with the number of dilated lysosomes and the increased number of LC3-positive puncta in RGC-5 cells. Rottlerin treatment (1 μM) inhibited EMB-induced vacuole formation and decreased the number of LC3-positive puncta. Scale bar, 50 μm.

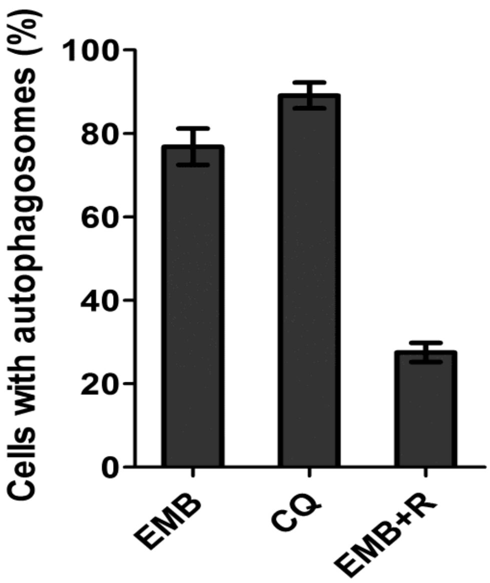

Supplementary Figure 3. Quantitative analysis of autophagosome puncta in RGC-5 cells. At least 100 cells were examined for each experimental group. Data shown are the means ± SEM of three independent experiments.
